# Broad ligament pregnancy – A rare and challenging diagnosis

**DOI:** 10.1002/ccr3.4823

**Published:** 2021-09-15

**Authors:** Paweł Sadłecki, Marek Grabiec, Małgorzata Walentowicz‐Sadłecka

**Affiliations:** ^1^ Department of Obstetrics and Gynecology Collegium Medicum in Bydgoszcz Nicolaus Copernicus University in Torun Bydgoszcz Poland; ^2^ Department of Obstetrics and Gynecology Bielanski Hospital Warsaw Poland

**Keywords:** abdominal pregnancy, broad ligament pregnancy, ectopic pregnancy, laparoscopy

## Abstract

Broad ligament pregnancy is a very rare life‐threatening form of ectopic pregnancy, in which implantation occurs within the peritoneal cavity. The advantages of a laparoscopic approach over a laparotomy in this setting include a reduced estimated blood loss, a shorter operating time, reduced analgesic requirements, shorter hospital stay, and convalescence.

## Graphical abstract

An early broad ligament pregnancy can be safely removed by laparoscopy. Ectopic localization of pregnancy typically has a detrimental effect on future fertility; a minimally invasive approach is advocated whenever feasible.

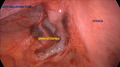

A 35‐year‐old woman (G3, 5+5 weeks) was referred due to suspected ectopic pregnancy. Transvaginal ultrasonography demonstrated a heteroechoic area in the left adnexal view (Figure 1). The patient did not consent to be offered conservative management (Methotrexate) and was qualified for diagnostic laparoscopy. An ectopic pregnancy was found at the base of the broad ligament (Figures 2, 3, 4). The ectopic tissues were removed and histopathological examination revealed the presence of compact decidua and villi (Video S1). If the condition is detected early, minimally invasive treatment is the preferred option as it does not negatively affect fertility and is sufficiently effective in such cases.^1^

**FIGURE 1:**
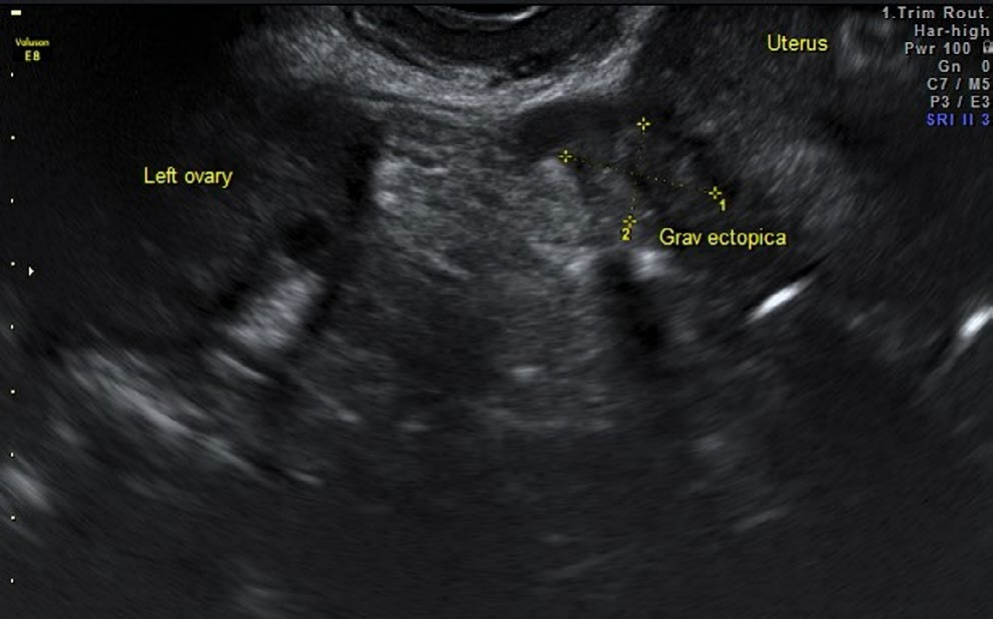
Transvaginal ultrasonography demonstrated a heteroechoic area in the left adnexal view that might correspond to an ectopic pregnancy

**FIGURE 2:**
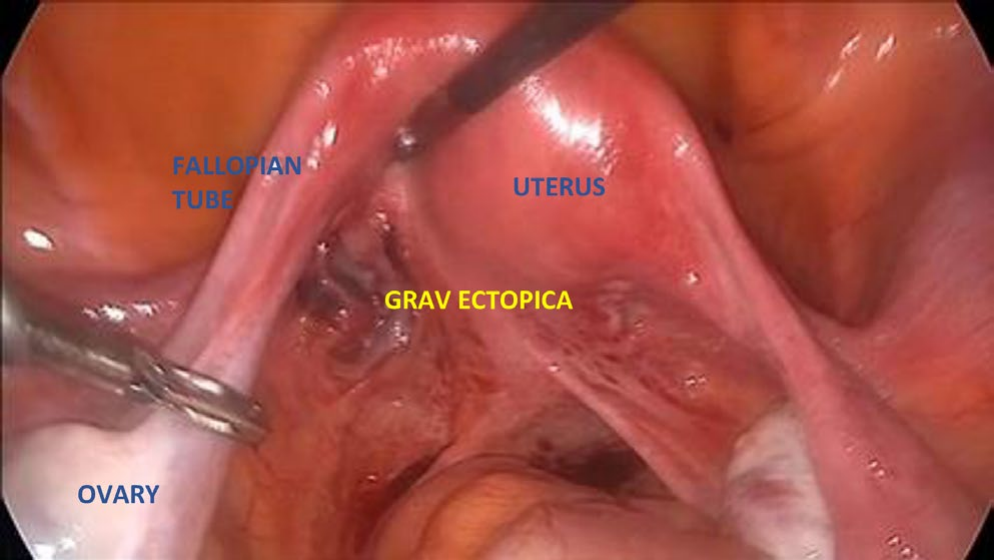
Ectopic pregnancy was found at the base of the posterior lamina of the broad ligament of the uterus. Both fallopian tubes remained unchanged

**FIGURE 3:**
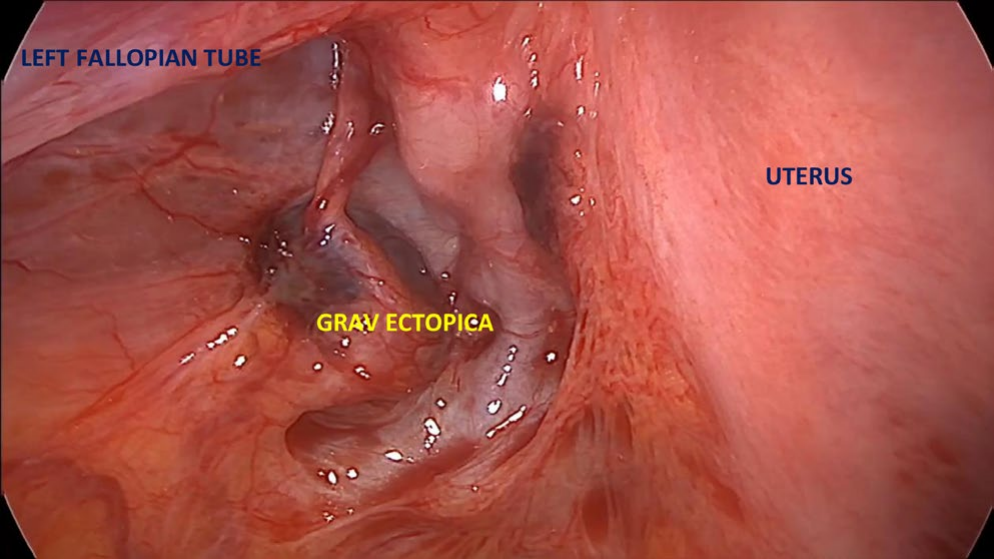
Abdominal pregnancy located at the base of the broad ligament

**FIGURE 4:**
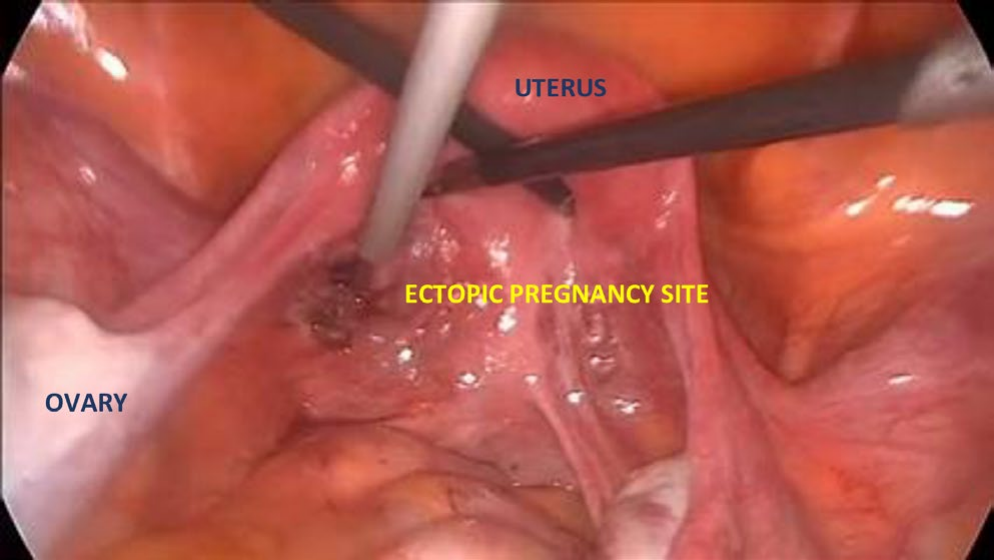
Ectopic tissues were removed carefully, and the bleeding sites were coagulated with a bipolar electrode

## CONFLICT OF INTEREST

The authors declare that they have no conflict of interest.

## AUTHOR CONTRIBUTIONS

PS: concept of the study, writing manuscript; MG: correction of the manuscript; MWS: writing and correction of the manuscript.

## CONSENT

Written and informed consent was sought from the study participant.

## Supporting information

Video S1

## Data Availability

The data that support the findings of this study are available from the corresponding author upon reasonable request. The study was approved by the Local Bioethics Committee at Collegium Medicum in Bydgoszcz, Nicolaus Copernicus University in Torun.
